# Connecting remote populations to public health: the case for a digital immunisation information system in Nunavut

**DOI:** 10.1080/22423982.2017.1358566

**Published:** 2017-08-06

**Authors:** Lindsay A. Wilson, Barry Pakes, Malia S. Q. Murphy, Katherine M. Atkinson, Cameron Bell, Kumanan Wilson

**Affiliations:** ^a^ Clinical Epidemiology Program, Ottawa Hospital Research Institute, Ottawa, Canada; ^b^ Dalla Lana School of Public Health, University of Toronto, Toronto, Canada; ^c^ Department of Public Health Sciences, Karolinska Institutet, Stockholm, Sweden; ^d^ Faculty of Medicine, University of Ottawa, Ottawa, Canada

**Keywords:** Mobile health, digital health, immunisation, vaccination, northern health, remote communities, public health

## Abstract

Despite the best efforts of local healthcare workers and health officials, Nunavut, a large geographical region in Northern Canada, has struggled with outbreaks of vaccine-preventable diseases (VPD). We contend that the implementation of an immunisation information system (IIS) could strengthen prevention and response efforts to this and future outbreaks of vaccine-preventable diseases. Developing an IIS in Nunavut that builds on the existing CANImmunize infrastructure would reduce the cost and complexity of developing a new IIS, and allow Nunavut to benefit from the ongoing efforts to secure data on the CANImmunize platform. Such a system would enable the identification of individuals and subpopulations at highest risk of infection based on vaccine series completion and permit the exploration of the underlying causes of outbreaks in the territory through consideration of demographic and temporal factors. Confirmed high rates of vaccination in the context of an outbreak would indicate potential issues with vaccine efficacy while low rates of vaccination would suggest that efforts should be devoted to increasing vaccine coverage. This approach could also lay the foundation for infrastructure expansion to other remote and/or Indigenous communities where geographical and accessibility issues complicate health care utilisation and monitoring, both in Canada and internationally.

## Introduction

Northern and remote communities present unique challenges when it comes to the implementation, monitoring, and evaluation of public health and health care programs.[1,2] This is true in the territory of Nunavut, a large geographical region in Northern Canada with a primarily Indigenous Inuit population. Despite the best efforts of local healthcare workers and health officials, Nunavut has struggled with outbreaks of vaccine-preventable diseases (VPD), including a recent prolonged pertussis outbreak that lasted more than a year and led to substantial morbidity and resource diversion.[3,4] A major contributing factor to the difficulty in identifying, responding to, and preventing outbreaks of VPDs is the absence of high-quality immunisation data, both at the individual and population levels. This gap in data constrains efforts to conduct case and contact management, as well as assessments of overall immunisation coverage.

The implementation of an immunisation information system (IIS) in Nunavut could help to address this gap and support the efforts of local health care workers and public health authorities. IISs are “confidential, population-based, computerised databases that record all immunisation doses administered by participating providers to persons residing within a given geopolitical area”.[5] At the population level, IISs provide aggregate data on vaccinations for use in surveillance and coverage assessments. We believe mobile technology could facilitate the implementation of IISs, provide an opportunity to address immunisation challenges experienced in Nunavut, and could serve as a model for other regions facing similar challenges. We present our vision for the development of a mobile-enhanced IIS in Nunavut that leverages the technology we utilised to develop Canada’s national mobile immunisation application, CANImmunize. Our approach will overcome the unique challenge of limited internet connectivity and cellular coverage and will provide immunisation data that can be utilised to prevent and respond to future vaccine-preventable disease outbreaks. Furthermore, it will ensure that public health practitioners and health care providers are equipped with the necessary data to best protect the public.

## Current challenges in collecting immunisation data

For many Northern residents, access to healthcare can be hindered by geographic isolation, inclement weather, and other factors.[6] These challenges are present in Nunavut, where the territory’s 37,000 residents are distributed across 25 small communities and 2,000,000 km^2^.[7] Some health centres in these communities are affected by staffing shortages and high rates of turnover.[8,9] Internet and communication services are improving, but frequent disruptions can impede health service delivery and health data monitoring.[10] Health indicators for the territory reflect these challenges; despite having the highest per capita health expenditures in Canada,[2,11,12] Nunavut has disproportionately high rates of infectious disease, chronic disease, and age-specific mortality.[10] Recent evidence indicates that infants in Northern Canada face some of the highest rates of lower respiratory infection in the world, and Inuit children in particular demonstrate greater susceptibility to such infections.[3,4] Reasons for this remain uncertain and more data are needed to guide public health officials.

In addition to and potentially exacerbated by these factors, data indicate that susceptibility to vaccine-preventable illnesses is of concern in Nunavut.[13,14–19] In the case of influenza vaccination, coverage varies significantly across communities, with one study suggesting that uptake ranged from 12–77% in 2007–2008.[20] Latest data from the 2013 Childhood National Immunisation Coverage Survey suggest that up-to-date coverage for diphtheria, pertussis, and tetanus (DPT) among children in Nunavut was more than 10% below national coverage rates, ultimately falling below 50% among 17-year-olds. Furthermore, Nunavut ranked lowest of all Canadian provinces and territories for up-to-date coverage estimates for Hib, MMR, and polio vaccination among children aged 2 and 7 years.[21]

While these data suggest that vaccine coverage rates in some communities are quite low, it is not clear whether vaccine uptake is truly low, or if these findings are instead a product of a lack of a consistent, systematic, and timely reporting system for immunisation in the territory. Given the seriousness of the recent pertussis outbreak,[22–24] understanding the cause of this outbreak and whether it stemmed from under-vaccination or waning immunity is crucial for preventing future outbreaks. Confirmed high rates of vaccination in the context of an outbreak would indicate potential issues with vaccine efficacy while low rates of vaccination would suggest that efforts should be devoted to increasing vaccine coverage. In particular, there is a clear need for greater emphasis on improving the accuracy of coverage estimates in Northern communities, especially those in rural and remote areas.

Further compounding the difficulty of developing accurate estimates of vaccine coverage and thereby increasing the effectiveness of outbreak response strategies is the absence of a territorial IIS in Nunavut. Immunisations in Nunavut are currently documented within patient charts, on hard-copy immunisation cards kept in health clinics and on health service utilisation forms. Each of these data recording systems has unique challenges with respect to the accuracy, timeliness, and usability of the information. Specifically, delays between patient interaction and receipt of vaccination data from the health utilization form by the Department of Health can be up to 8–12 months, impeding the capacity for timely reporting and potentially impacting the development of strategies for addressing disease outbreaks. In the case of the pertussis outbreak, the absence of a digital record of immunisation resulted in healthcare providers having to manually search patients’ charts in order to identify individuals who had not been vaccinated, a difficult and time-consuming process. In the capital city of Iqaluit, an electronic medical record is being used to record immunisations, but the interface being used and the data being collected cannot easily be used for clinical or public health purposes. An IIS could make this identification process much more accurate and efficient in equipping health care providers with the information they need.

## Opportunities created by digital technology

A mobile-enhanced IIS would provide opportunities for real-time recording of immunisations with improved accuracy and efficiency over commonly used paper-based alternatives. This, in turn, would facilitate the monitoring of vaccine coverage and allow for the development of tailored interventions to improve vaccine uptake and outbreak response.

Beyond providing a means for comprehensive assessments of vaccination coverage, a mobile-enhanced IIS into which vaccination data may be directly recorded would provide a sustainable solution to Nunavut’s need for a functional IIS. Such a solution would allow Nunavut to “leapfrog” other currently used approaches to vaccine monitoring, with substantial potential value to the territory as a whole:

Population-based centralized immunization registries are a solution to overcoming linguistic and cultural barriers hindering completion of health surveys in high-risk population groups. Moreover, computerized records could facilitate the work of health professionals in assessing the immunization status of patients and issuing recall-reminders for those who are eligible or overdue for vaccine administration. (p. e271) [1]

A mobile-enhanced IIS for Nunavut would enable local health centres and public health authorities to directly input vaccination data into the digital platform, facilitating tracking of vaccination dosages administered throughout the territory. This would improve the efficiency of vaccine delivery, allow for accurate and seamless maintenance of vaccination histories, facilitate timely immunisation, improve outbreak response, and target resource allocation to at-risk individuals or underserved communities.

## Designing a digital immunisation information system for Nunavut

Opportunities exist to leverage existing infrastructure to support the creation of a mobile-enhanced IIS. CANImmunize is a free mobile application that provides Canadians with modern digital tools to help manage their families’ immunisation records and access up-to-date and reliable information on vaccination in Canada. The CANImmunize project has recently received funding from the Public Health Agency of Canada to further develop the platform to include automatic backup functionality in a secure and centralised database, improved forecasting of provincial and territorial immunisation schedules, and platform maintenance. Developing an IIS on top of the existing CANImmunize infrastructure would reduce the cost and complexity of developing a new IIS, and allow Nunavut to benefit from the ongoing efforts to secure data on the CANImmunize platform.

**Figure 1.**
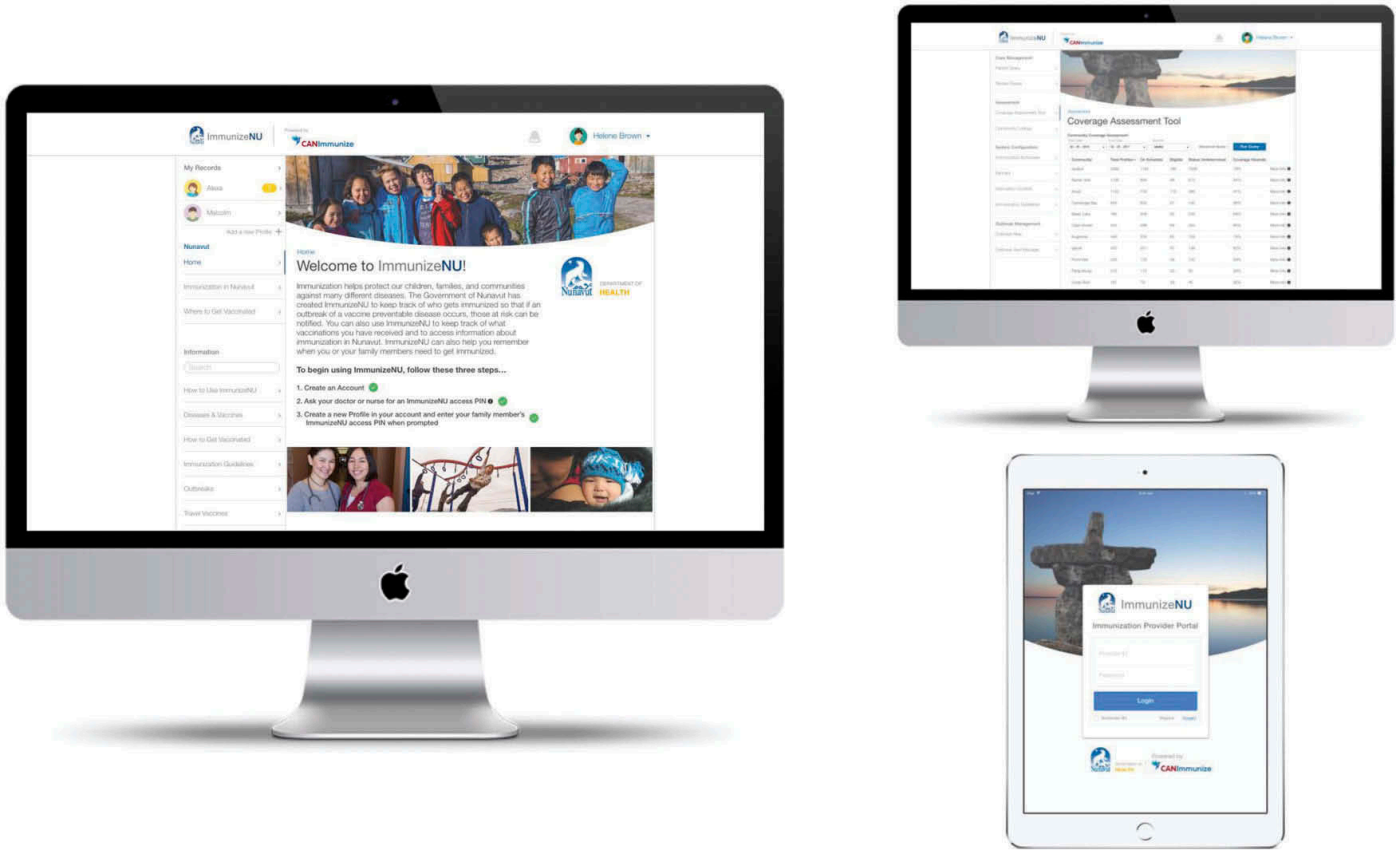
Sample mock-ups for Nunavut platform of CANImmunize.

The secure data storage infrastructure developed for CANImmunize could be leveraged to facilitate the storage of Nunavut’s immunisation records. While CANImmunize relies on families to input their immunisation data, we propose to develop a suite of tools to be used by local healthcare providers, including a custom web portal and mobile application interface for capturing or entering immunisation records for inclusion in a centralised database. Healthcare providers would record data elements including user age, sex, community, and date of vaccination, and antigens covered, and this information would be made available to authorised Nunavut public health officials in order to perform coverage assessments. Importantly, this approach would take into consideration the limited internet capabilities of many Nunavut communities. A tablet application would be developed that would enable health centre staff (e.g. nurses and public health nurses) to record immunisations for local communities even when offline. Data would be stored within the mobile application and synchronised with the centralised database once an internet connection is re-established. Using this approach, public health authorities would still be able to collect immunisation data even for communities with minimal internet access. Mock-ups of the Nunavut applications are provided in Figure 1.

It will also be important to address issues concerning data security and privacy, as well as cultural issues concerning the collection of data from Indigenous peoples. While CANImmunize is highly secure and compliant with legislation such as Ontario’s Personal Health Information Protection Act, its primary use to date has been the storage of immunisation records from the public with their consent. Our proposed use of CANImmunize to help providers and public health officials store immunisation information is akin to providing a clinician with an electronic medical record, and as such falls under a separate privacy/consent framework which will require further privacy impact assessment. Perhaps the most significant consideration in implementing this system would be sensitivity to cultural issues related to data collection. Previous research with Indigenous communities has at times been exploitative and has not benefited Indigenous communities, contributing to issues of mistrust between Indigenous communities and non-Indigenous researchers.[25] Ensuring that this work meets the needs of and is authorised by the people of Nunavut will be a key component of this work. Engagement with the local population to ensure that all collection and use of this data would serve the best interests of the communities while remaining respectful of cultural norms and traditions surrounding public health data collection will be essential.

**Figure 2.**
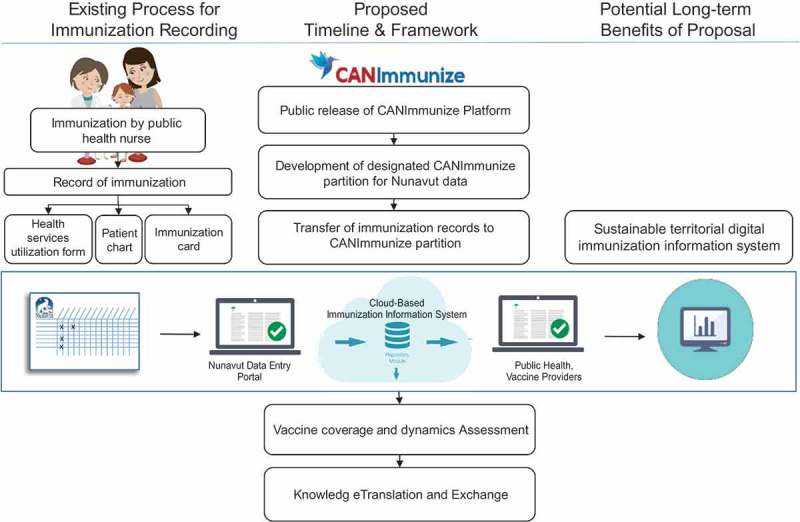
Schematic describing existing immunisation record process and proposed CANImmunize-based electronic system.

We envision that our proposed mobile-enhanced IIS would satisfy two main user requirements: the need to capture immunisation data and the need to extract immunisation data. To capture immunisation data, the web and mobile interfaces would enable nurses to input immunisation information as the immunisations are administered in real-time, and data entry clerks to input archival data contained on existing paper-based records currently in the possession of the Nunavut Department of Health. Additionally, data extraction and analysis would be performed by Nunavut health officials through the web portal. Functions such as patient look-ups and data exports could be conducted to perform coverage assessments and surveillance. A visual summary of the current process for immunisation data collection in Nunavut is presented in Figure 2, along with how the proposed work will leverage the CANImmunize platform to perform a coverage assessment in the territory.

## Benefits of a digital solution

Given recent outbreaks of vaccine-preventable disease and community and health centre mobilisation around outbreak response, members of the Government of Nunavut Department of Health engaged The Ottawa Hospital (the developers of CANImmunize) to collaboratively develop an IIS tailored to the unique needs of Nunavut. Nunavut communities and public health providers are enthusiastic and engaged around the idea of immunisation as a solution to certain vaccine preventable disease outbreaks and the collateral challenges they impose on the health care infrastructure. The potential knowledge generated by an initiative of this kind would allow for this enthusiasm to translate into more accurate estimates of vaccine coverage that could help to prevent future outbreaks and tailor response strategies. Ongoing engagement between The Ottawa Hospital, the Government of Nunavut Department of Health, nursing staff, and community members will be needed to ensure that this project is respectful to the needs and culture of the local populations and of maximum benefit to those who will be most affected.

There are numerous potential benefits that could arise from the implementation of a mobile-enhanced IIS in Nunavut (Table 1). Such a system would enable the identification of individuals and subpopulations at highest risk of infection based on vaccine series completion and permit the exploration of the underlying causes of un- and under-vaccination in the territory through consideration of demographic and temporal factors. As a result, variation in up-to-date coverage and age-appropriate coverage by geographic region (community of residence), age, and sex could all be assessed. Furthermore, analysing variations in immunisation coverage across geographic regions and demographic subpopulations would enable identification of possible determinants of vaccination coverage. This information could be used to tailor interventions toward specific individuals or communities, target human resource efforts toward underserviced communities, modify immunisation schedules or strategies, and strengthen outbreak response through active case and contact management.

**Table 1.** Vaccination challenges and opportunities for improvement.

Challenge	Opportunity for a digital IIS
Capturing and recording vaccinations administered Current documentation practices require manual data entry of paper records to electronic systems Lack of a territory-wide electronic reporting system for immunisations Medical care often occurs outside of home community, making it difficult to track vaccines administered	Immunisations can be recorded in real-time via an IIS Cloud-based applications allow health care providers to input data regardless of location and without delays Allows healthcare providers to rapidly analyse immunisation data to inform delivery of care
Calculating vaccine coverage and directing public health efforts accordingly Rapidly assessing vaccine coverage in different communities and geographical areas requires data analysis and this causes delays in producing information to guide public efforts	Jurisdictions with low vaccine coverage can be identified Data visualisations identifying pockets of un-immunised as well as under-immunised populations in addition to more detailed quantitative reports Public health officials can initiate campaigns aimed at increasing vaccine uptake in under-immunised communities or demographic sub-groups
High rates of vaccine-preventable diseases Young population Limited access to health services Disparities stemming from social determinants of health (e.g. poverty and social exclusion)	Delivering interventions which improve vaccine coverage Provides infrastructure for tailored, digital interventions to be delivered to patients at a low cost Vaccine-preventable illnesses can be reduced and managed as a result of broader immunisation coverage Vaccine schedules and recommendations can be readily communicated to health care providers
Limited Internet connectivity Disruptions in health service delivery	Mobile platform permits data collection even without an internet connection Information entered offline will synchronise with central database after connection is re-established
Outbreak management Difficulty in establishing cause of outbreak (i.e. vaccine failure vs. inadequate coverage) Issues with contact management when coverage is unclear	Ability to rapidly assess vaccine coverage data permits public health officials to respond more quickly and effectively to outbreaks Cause of outbreak can be more readily ascertained and case-management facilitated Accurate collection and reporting of vaccine adverse events can be utilised to ascertain vaccine safety and effectiveness

Vaccine coverage could be calculated overall for Nunavut, as well as by community and administrative region. Temporal trends could also be considered through the analysis of date and season of vaccination and the proportion of “off-schedule” immunisations. By comparing patients’ dates of vaccination to Nunavut’s recommended vaccine schedule, the role of local catch-up programs in immunisation could be better understood. This could provide insight into different models of vaccine delivery within health centres and schools, facilitating sharing of best practices and offering valuable, relevant information that could be provided to parents about the benefits of participating in such campaigns. It could also help to determine whether standard vaccination opportunities are being missed and what impact these catch-up campaigns have on overall coverage. These data could serve to identify the highest and lowest performing communities for vaccination coverage, enabling the identification of possible determinants of vaccination coverage.

The difficulties associated with monitoring vaccine uptake in Nunavut may be generalisable to other jurisdictions as well, particularly those in remote regions, both in Canada and elsewhere. Any region where geographical and other accessibility issues hinder access to immunisation uptake and monitoring could derive substantial benefit from a mobile-enhanced IIS. Successful implementation of a mobile-enhanced IIS could pave the way for future innovations in vaccine surveillance across Northern Canada. Importantly, the potential knowledge from an initiative of this kind could inform future partnerships between the Department of Health and Northern communities to deliver targeted interventions that are culturally appropriate and acceptable to the Indigenous communities of Nunavut.

## Conclusion

Here we have identified an opportunity to establish a sustainable solution to provide Nunavut with a functional IIS that would provide substantial benefit to both patients and healthcare providers. A territorial mobile-enhanced IIS could be used to reliably and efficiently identify under- or un-immunised communities in Nunavut in real-time. As described above, generating accurate and reliable coverage estimates is particularly challenging in Nunavut. A strategic partnership between academic researchers, local healthcare providers, public health authorities, and local communities could provide both a comprehensive assessment of coverage of recommended childhood vaccinations in Nunavut and a digital information system for future storage and monitoring of vaccine records.

If a real-time, accessible platform can be successfully developed and implemented in Nunavut, the implications for other rural and remote communities are clear. A mobile-enhanced IIS emerging from Nunavut could lay the foundation for infrastructure expansion to other remote and/or Indigenous communities where geographical and accessibility issues complicate health care utilisation and monitoring, both in Canada and internationally. Further exploration of this potentially promising intervention is needed. Canadian population health surveys often lack specific data regarding the health of First Nations, Métis, and Inuit populations, rendering policy, programme, and practice-based decision-making in Indigenous communities very difficult. In 2012, the First Nations and Inuit Health Branch (FNIHB) Strategic Plan was launched to improve the availability and accessibility of high quality data relating to Indigenous health. The strategic partnerships that could be developed through the initiative would represent an exciting step toward achieving this objective.
